# First evidence of clinical protection from an EEHV1A mRNA vaccine in naturally infected juvenile elephants

**DOI:** 10.1128/jvi.02186-25

**Published:** 2026-07-28

**Authors:** Michael Wenninger, Lauren Farris, Jessica Heinz, RongSheng Peng, Gary S. Hayward, Jessica Watts, Jeroen Pollet, Jenny Nollman, Paul D. Ling

**Affiliations:** 1 Cincinnati Zoo and Botanical Garden 57701, Cincinnati, Ohio, USA; 2 Department of Molecular Virology and Microbiology, Baylor College of Medicine 189531, Houston, Texas, USA; 3 Viral Oncology Program, Johns Hopkins School of Medicine 1500 , Baltimore, Maryland, USA; 4 Department of Pediatrics, Division of Tropical Medicine, Baylor College of Medicine 506057, Houston, Texas, USA; Dartmouth College Geisel School of Medicine, Hanover, New Hampshire, USA

**Keywords:** vaccine efficacy, hemorrhagic disease, mRNA vaccine, elephant endotheliotropic herpesvirus, Asian elephant

## Abstract

Juvenile Asian elephants who have lost maternal antibodies are particularly susceptible to fatal hemorrhagic disease caused by primary infection with elephant endotheliotropic herpesvirus 1A (EEHV1A). To address this vulnerability, a multi-antigen EEHV1A mRNA vaccine has been administered to EEHV1A seronegative calves at four institutions. However, the extent to which this vaccine confers protective immunity remains an open question. In November 2023, two juvenile male calves (aged 6 and 7 years) were transferred from Dublin Zoo to the Cincinnati Zoo and Botanical Garden and confirmed to be seronegative for EEHV1A. Both calves received an experimental EEHV1A mRNA vaccine in October 2024, followed by a booster dose in November. Post-vaccination serological responses were robust, with significant antibody production against vaccine-encoded glycoproteins (gB, gH, gL, and gO), although titers started to decline by day 102 post-booster. In February 2025, the younger calf developed EEHV1A viremia, followed by the older calf in March. Both maintained viral loads below 6,000 viral genome equivalents (VGE)/mL and exhibited no hematological abnormalities or clinical signs of disease. Serum collected post-viremia revealed sustained antibody titers against the four vaccine antigens, approaching levels observed in naturally infected elephants. Despite confirmed viremia, both calves remained clinically healthy, and viral loads resolved without therapeutic intervention. These findings suggest that immunity generated against the EEHV proteins encoding the conserved herpesvirus attachment fusion complex can confer protective immunity and mitigate disease severity following primary infection in juvenile elephants.

**IMPORTANCE:**

This study delivers an early demonstration of applying mRNA vaccination to a herpesvirus outside conventional laboratory models and, to our knowledge, the first such use in an endangered mammalian species. EEHV-HD is the leading cause of death in juvenile Asian elephants, a species critical to global biodiversity and cultural heritage. Our findings provide both immunological evidence and real-world clinical protection, marking a significant advancement in wildlife health and expanding the translational potential of mRNA platforms beyond human medicine. These studies address an urgent conservation challenge and introduce a novel antiviral strategy with broad implications for herpesvirus vaccine development.

## INTRODUCTION

Elephant endotheliotropic herpesvirus-associated hemorrhagic disease (EEHV-HD) is the leading cause of mortality in juvenile Asian elephants and represents a major threat to the long-term sustainability of captive and wild populations (1, 2). Among the three major EEHV types endemic to Asian elephants, each comprising A and B subtypes, EEHV1A is most frequently associated with fatal hemorrhagic disease in young individuals.

Herpesvirus infections typically progress through several stages (3). The initial stage is primary infection, which may be subclinical or present as localized or systemic lesions. Following the development of an adaptive immune response, comprising both humoral and cellular components, the infection is brought under control, and the virus establishes latency in cells or tissue types specific to each herpesvirus. Under normal circumstances, the latent virus can periodically reactivate, often resulting in viral shedding from mucosal surfaces.

In elephants, most adults previously infected with EEHV shed virus intermittently, most commonly detected in trunk secretions, with shedding frequency and viral load likely influenced by environmental conditions and individual genetic factors (1, 4–6). In cases of EEHV primary infection, survivors frequently shed large quantities of virus in trunk secretions for weeks or even months following recovery, before transitioning to the intermittent shedding pattern observed in EEHV-experienced elephants (1, 4–6).

Serological and qPCR surveillance of mucosal secretions suggest that most adult Asian elephants have been exposed to all three major EEHV types found in this species (EEHV1, EEHV4, and EEHV5) (6–11). Maternal antibodies are transferred *in utero* and possibly through milk, but in the absence of a primary infection, these antibodies typically decline to low or undetectable levels by 24–36 months of age (12–16). During this period, some calves remain completely naive, while others acquire infection with one or more EEHV types before maternal antibodies have waned (12, 13, 15, 16). Importantly, there is no strong evidence that prior infection with one EEHV type confers cross-protection against disease caused by other types. When antibody titers fall below a protective threshold for a given EEHV type, elephants become susceptible to clinical illness associated with a primary infection, which can lead to rapid-onset, often fatal hemorrhagic disease, particularly in seronegative animals. Most cases of EEHV-HD are caused by EEHV1A and, to a lesser extent, EEHV1B, although EEHV4 and EEHV5 have also been implicated (1, 8, 11, 17).

While young elephants become vulnerable to EEHV hemorrhagic disease as maternally derived antibodies decline, the underlying mechanisms of viral spread and tissue injury are still not fully defined. Current evidence suggests that initial exposure often occurs through intermittent or intensified viral shedding from herd members, including animals actively recovering from or experiencing EEHV-associated illness (1, 2, 6, 10). Although the earliest sites of viral replication remain uncertain, some findings indicate that monocyte-macrophage lineage cells may play a role in distributing the virus throughout the body. Pathologic examinations consistently highlight extensive injury to vascular endothelial cells, including characteristic intranuclear inclusions that reflect direct viral damage (2). Many fatal cases also show microthrombi in multiple organs, particularly the lungs, alongside widespread hemorrhage and marked thrombocytopenia, patterns that align with a disseminated coagulopathy during active infection. Ultimately, death appears to result from severe, diffuse vascular compromise leading to multiorgan failure, with acute myocardial hemorrhage commonly noted. In several affected calves, a pronounced inflammatory response has been documented even in the absence of detectable bacterial infection, raising the possibility that an overwhelming cytokine surge may contribute to disease severity (2).

Recent advances in EEHV surveillance, including EEHV1-specific serological assays (7, 12) and quantitative PCR (qPCR) for viral DNA detection (6, 18), have improved the ability to determine serostatus and monitor viremia or viral shedding, thereby informing clinical management strategies. The development of an ELISA targeting the EEHV1A gH/gL/gO trimeric complex has further refined serological assessment by enabling the detection of conformational epitopes not captured by individual antigens in LIPS assays (19).

To address the urgent need for preventive measures, a multi-antigen mRNA vaccine encoding EEHV1A glycoproteins B, H, L, and O was developed to elicit protective immunity and reduce the risk of EEHV-HD (20). Codon-optimized sequences for the four antigens were synthesized, individually encapsulated in lipid nanoparticles, and combined. In murine models, two doses of the vaccine induced robust humoral and cellular immune responses without adverse reactions. Subsequently, nine Asian elephants—three seropositive and six seronegative—were vaccinated with two doses of the 400 μg multiantigen mRNA vaccine separated by 3 weeks and monitored for antibody responses over a 6-month period (19). Vaccination led to increased antibody titers in both previously exposed and naive elephants, although the extent of protective immunity remains to be fully characterized.

In this report, we describe the transfer of two juvenile Asian elephants from the Dublin Zoo to the Cincinnati Zoo & Botanical Garden, their integration into the resident herd, administration of the experimental EEHV1A vaccine, and the outcome following apparent primary infection with an EEHV strain likely shed by one or more herd mates.

## MATERIALS AND METHODS

### Animals, sample collection, and processing

Three elephants are described in this study: (i) a 38-year-old adult male (AM), (ii) a 6-year-old male (elephant 5), and (iii) a 7-year-old male (elephant 6). Elephants 5 and 6 are the same as described in a related study (19). The EEHV1A mRNA vaccine targeting glycoproteins gB, gH, gL, and gO was prepared as previously described (20). Briefly, mRNAs encoding each glycoprotein from the EEHV1A Kimba strain (KC618527) were synthesized by the Methodist RNA Therapeutics Center (Houston, TX) and individually encapsulated in lipid nanoparticles (LNPs) under sterile conditions, followed by filtration through a 0.22 µM filter. Equal amounts (100 µg each) of the four LNP-formulated mRNAs were combined to create a 400 µg total dose in approximately 2 mL of phosphate buffer with 8% sucrose. All vaccines were frozen and stored at −80°C until use. Each elephant received two intramuscular injections of the 400 µg vaccine. Elephants 5 and 6 received their first dose on October 21, 2024, while elephant 5 received a booster dose on November 13, 2024, and elephant 6 on November 11, 2024.

Whole blood and trunk-wash samples were collected from each elephant as previously described (6). Briefly, 2 mL of blood was drawn from the auricular vein into EDTA-coated tubes and stored at 4°C until DNA extraction, which was performed within 24 h of collection. Typically, 200 μL of blood was processed for DNA, with the remaining volume stored at –80°C. Trunk-wash samples containing nasal secretions were collected using the USDA-recommended protocol for *Mycobacterium tuberculosis* surveillance. In brief, 50 mL of sterile 0.9% saline was instilled into each naris, and the proboscis was elevated for 20–30 s. Elephants were then instructed to expel the fluid into a sterile 1-gallon plastic freezer bag. The effluent was transferred to a sterile 50 mL conical tube and kept on ice until processing. Samples were centrifuged at 1,500 × *g* for 10 min; supernatants were discarded, and cell pellets were stored at –80°C until DNA extraction.

### qPCR, hematology, and serology

DNA extraction from blood and trunk wash samples for quantitative PCR (qPCR) or conventional PCR for EEHV1A detection was performed as previously described (6, 18). Complete blood counts (CBCs) were conducted using standard veterinary hematology protocols and included manual white blood cell differential counts and platelet estimates. Reference ranges were based on CBC values collected in the 14 months prior to viremia and calculated as the average plus/minus the standard deviation. EEHV-specific serology was performed using both luciferase immunoprecipitation system (LIPS) (12) and enzyme-linked immunosorbent assay (ELISA) platforms, including assays targeting the EEHV1A gH/gL/gO trimeric complex (19).

### Strain typing

Trunk-wash DNA was first screened for EEHV1A by qPCR. Five microliters of EEHV1A-positive DNA from the resident adult bull (AM) and from elephants 5 and 6 post-viremia were amplified using the REPLI-g kit (Qiagen) in a 20 μL reaction. One microliter of the REPLI-g product was used for conventional PCR amplification and sequencing.

Target loci included POL, vGPCR, U71-gM, and TK-gH, using previously published primers and conditions (21). The entire open reading frames for gO and gL were amplified and sequenced by a single round of PCR from trunk wash DNA with the following primers: gO: OPL2207 5′-CATTTCATTGATCATATGGACGCTT-3′ and OPL2208 5′-CATCTCTTCCTTCCTCAGCATAC-3′ and for gL: OPL 2193 5′-GTCAGTCAACCCAGCACTATC-3′ and OPL2194 5′-CGGGGTTTAATCGCCATTTAGTG-3′. Glycoproteins B and H required the first round PCR of partial regions of the open reading frame and the second round overlapping PCR products within each of the larger first round PCR products. Primers for gB include first round, OPL2217 5′-ATTCGGGAATGTGGACTGTATC-3′ and OPL2223 5′-GTCTACGTCTCGCAGCTTTATG-3′, followed by second round internal primers that amplify smaller overlapping segments of the first round product. Second round primer pairs were OPL2213 5′-ATTCGGGAATGTGGACTGTATC-3′ and OPL2224 5′-TCCTTGACTTCCCACGCAAAT-3′ and OPL2219 5′-CTGTACGACGGTCAACTGTATCG-3′ and OPL2223. The rest of the gB sequence was determined by first-round PCR sequencing with primers OPL2221 5′-TGCCGCACTGTCAGCTATATT-3′ and OPL2216 5′-AAGCCATCATCTACCGTAACTAT-3′. Initial sequencing of the TK-gH locus indicated that the Cincinnati strain was an identical match to a partial gH sequence (KM458130.1) and almost identical to the gH encoded by Umesh (OR543011). PCR primers to confirm the entire gH open reading frame were designed using these related gH sequences. In order to determine the rest of the gH open reading frame sequence, we employed two additional primer pairs that amplified overlapping sequences with each other. The pairs were OPL 2241 (fwd) 5′-ATACTTTGGCCGCTGTAGA-3′ and OPL 2248 (rev) 5′-TATACCCGTGGGCATATGTT-3′ and OPL2243 (fwd) 5′-GCCCAGGGTATATGACAAAG-3′ and OPL2236 (rev) 5′-TCACCTTGACGAGAACAGGAAA-3′.

PCR products were verified by gel electrophoresis, purified, and submitted for Sanger sequencing (GeneWiz/Azenta) along with the corresponding primers used to generate them. Sequence data were assembled and aligned to reference EEHV1A genomes using MacVector software. Strain characterization was carried out by Blast searches comparing different PCR sequences with existing sequences in GenBank and designated according to the labeling schemes described previously (21–25).

## RESULTS

**Fig 1:**
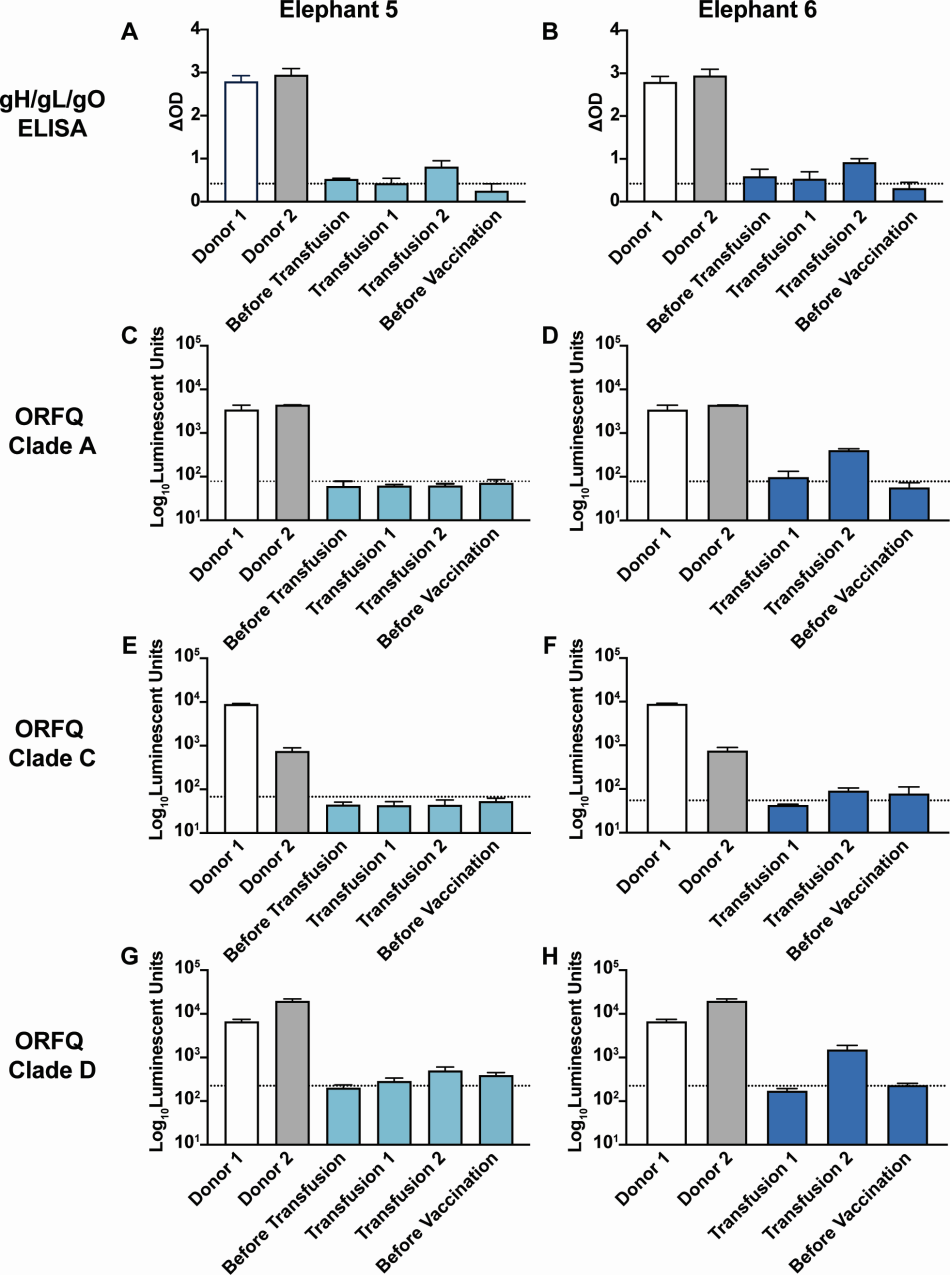
EEHV serostatus of elephants 5 and 6 prior to and after plasma transfusions. Antibody levels reactive against the gH/gL/gO trimeric complex upon arrival at the Cincinnati Zoo and Botanical Gardens and after two independent plasma transfusions (**A and B**). Antibody titers of the plasma donors are also shown. Each bar represents the average of two technical replicates from two separate experiments. The cutoff was determined by the receiver operating characteristic curve. Antibody titers reactive against three ORFQ proteins representing the major clades of EEHV1A strains were determined by luciferase immunoprecipitation system (LIPS) (**C–H**). Elephant 6’s pretransfusion sample was omitted due to insufficient volume. Data is reported as luminescent units on a log_10_ scale. Values shown are the average of two technical replicates and are representative of two independent experiments. The limit of detection was determined by the average of negative controls plus five standard deviations.

In November 2023, two juvenile male Asian elephants designated as elephants 5 and 6 (19), along with their dams, were transferred to the Cincinnati Zoo and Botanical Garden. Antibodies against ORFQ proteins representing nearly all EEHV1A strains (clades A, C, and D) have previously been used to detect prior EEHV1A infection (12, 26). Similarly, the gH/gL complex has served as a serological marker (7, 13); however, in this study, we also employed a gH/gL/gO trimer (19). Initial serological testing using both biomarker sets indicated that both calves were EEHV1A seronegative or at the assay cutoff (Fig. 1A through H).

Because all members of the resident herd were EEHV1A-seropositive (unpublished observations), plasma transfusions from seropositive donors were administered to the calves to provide passive immunity before and after herd introduction. This strategy was based on reports that transfused antibodies can persist for several months post-administration (Crooks and Suedmeyer, 2024 Global EEHV Symposium). Sero profiles of plasma donors, donors 1 and 2, were also assessed (Fig. 1A through H).

Antibody levels following the first transfusion could not be evaluated due to the absence of samples collected in the immediate post-transfusion period. After the second transfusion, both elephants exhibited detectable circulating antibodies against EEHV1A-specific biomarkers gH/gL/gO in the ELISA (Fig. 1A and B). In addition, elephant 5 exhibited a small increase in reactivity against only clade D ORF Q (Fig. 1G), while elephant 6 exhibited reactivity against all ORF Q clades (Fig. 1D, F, and H). These antibody levels subsequently declined to near baseline or undetectable levels in both LIPS and ELISA assays prior to vaccination (Fig. 1A through H) (19). Thereafter, elephants 5 and 6 were vaccinated with an experimental EEHV1A mRNA vaccine and received a booster dose 3 weeks later (19).

### Vaccination against EEHV appears to provide protection from EEHV-HD

**Fig 2:**
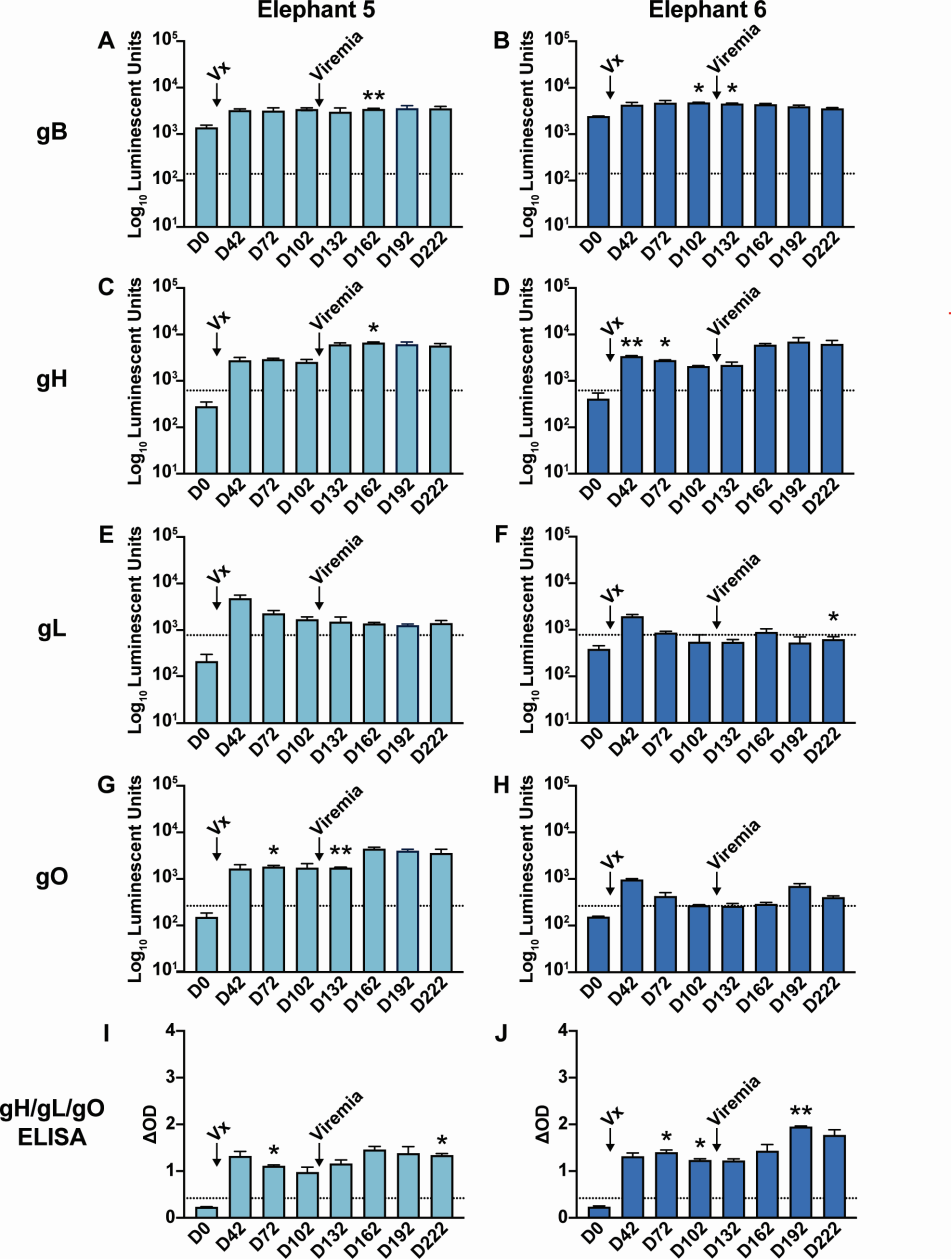
Humoral response to EEHV1A in two juvenile Asian elephants following mRNA vaccination and primary infection. Antibody levels to individual antigens included in the EEHV1A mRNA vaccine were determined by LIPS for gB (**A and B**), gH (**C and D**), gL (**E and F**), and gO (**G and H**). Arrows indicate approximate timing of vaccinations and onset of EEHV1A viremia. Values shown are two technical replicates and are representative of at least two independent experiments. Antibodies against the gH/gL/gO trimer protein were assessed by indirect ELISA (**I and J**). Each bar represents the average of two technical replicates from two separate experiments. Significance relative to day 0 (D0) was identified through a repeated measures one-way ANOVA. *P < 0.05, **P < 0.01.

**Fig 3:**
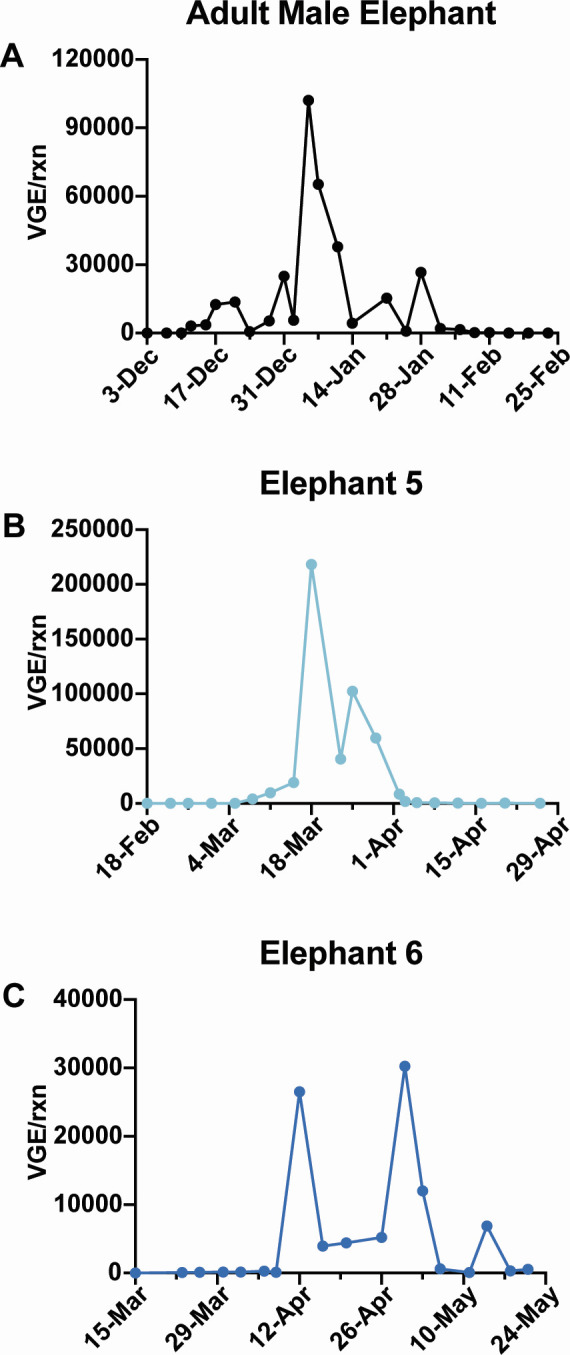
Trunk shedding in AM and elephants 5 and 6 around primary EEHV1A infection. Viral genome equivalents (VGE) per reaction (rxn) were plotted over time for a resident adult male (**A**) and vaccinated juveniles (**B and C**) before, during, and after primary viremia.

**Fig 4:**
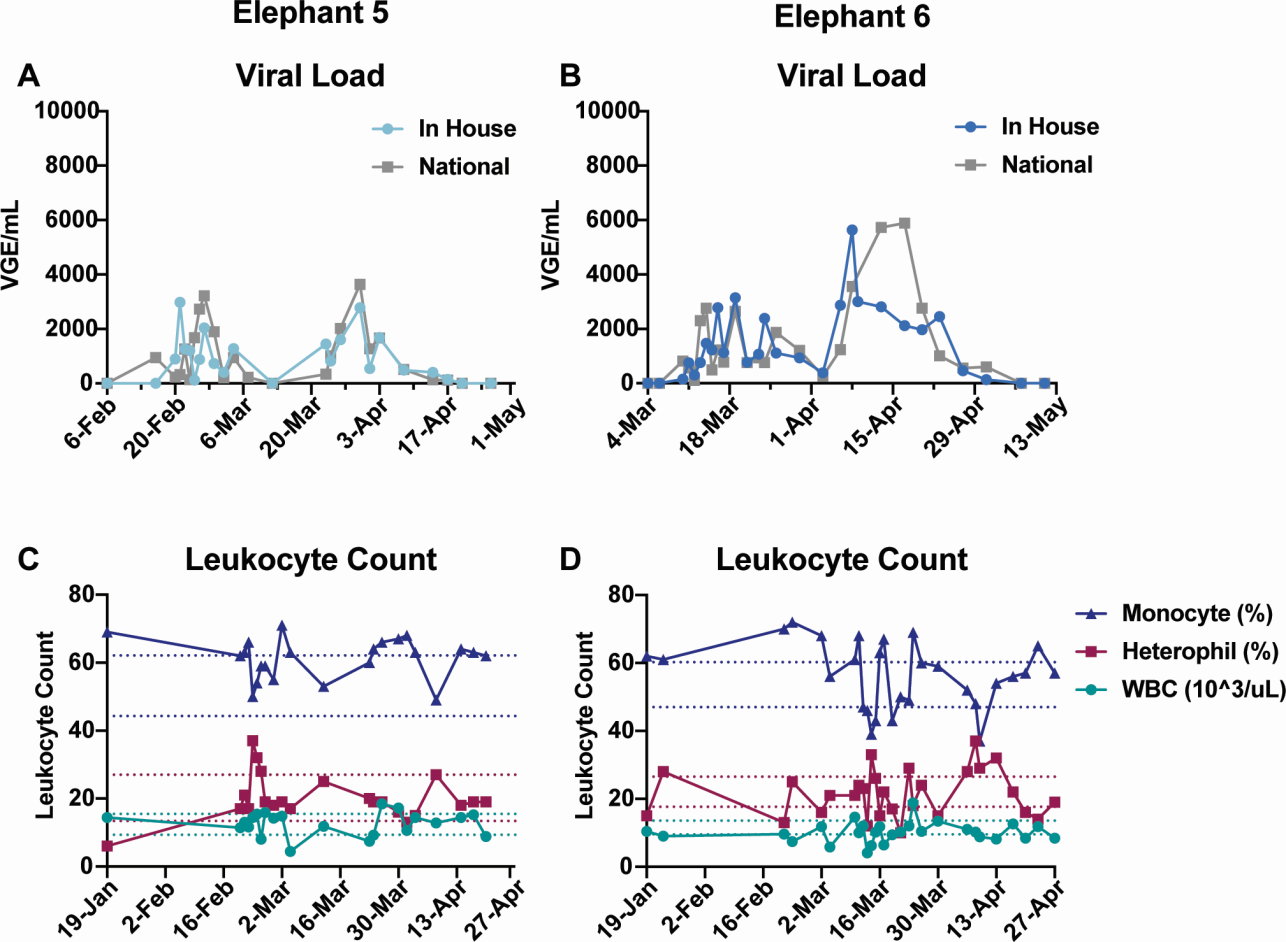
EEHV1A viral load and CBCs before, during, and after EEHV1A viremia. EEHV viral genome equivalents were quantified per milliliter of blood (y-axis) and plotted over the indicated time points on the x-axis (**A and B**). The plots represent numbers calculated from qPCR performed in two independent labs at the Cincinnati Zoo (blue dots) and the National Elephant Herpesvirus Laboratory (gray squares). White blood cell (green), heterophil (red), and monocyte (blue) counts were plotted during EEHV viremias with dotted lines corresponding to the normal reference range for each, respectively (**C and D**).

Serial serological monitoring following vaccination of these two elephants revealed increased antibody titers against gH, gL, gO, and a trimeric complex of these glycoproteins, with levels remaining above the assay cutoff through 102 days post-vaccination (Fig. 2C through J). Increased titers were also observed for gB, although baseline titers were already high prior to vaccination (Fig. 2A and B). gB is highly conserved among EEHV types, and the starting titers are likely from cross-reacting antibodies from previous infections with EEHVs 4 and/or 5 (12, 13). During this period, intermittent EEHV1A shedding was detected in trunk washes from the resident bull, AM, who is also known EEHV1A seropositive, with a prolonged shedding episode occurring in December 2024 through January 2025 (Fig. 3A). In February 2025, the younger calf, elephant 5, developed EEHV1A viremia, followed by elephant 6 in March 2025. Both elephants maintained low viral loads and exhibited no sustained significant deviations in white blood cells or heterophil or monocyte differential indices relative to their established individual baselines (Fig. 4C and D). A decrease in the monocyte:heterophil ratio is used as an important prognostic indicator for EEHV (11, 27). Both elephants exhibit a dip in this ratio coinciding with peak viremias, particularly notable for elephant 5 in February and April (Fig. 4A and C) and for elephant 6 in March and April (Fig. 4B and D) that immediately rebounded. Calves were monitored continuously by caretaker staff for signs of discomfort or changes in behavior, appetite, or activity. Veterinarians performed visual examinations daily to assess for edema, ecchymoses, or petechiation, increases in heart or respiratory rates, discomfort, or loss of condition. No clinical signs of EEHV-HD were observed, and primary viremia resolved spontaneously without therapeutic intervention. Both calves had detectable EEHV1A shedding in their trunk secretions during and after control of their viremias, which declined to undetectable or near undetectable levels in May 2025 (Fig. 3B and C).

### EEHV mRNA vaccine is effective despite genetic divergence by infecting strain

EEHV1A subtypes exhibit considerable genetic divergence across several loci, including U48 (gH-TK), U51 (vGPCR), U38 (POL), and U71 (gM), which are commonly used for subtype differentiation (6, 21–25). Strain-level characterization of the EEHV1A shed by the resident bull had not been determined previously, but subsequent DNA sequencing of trunk wash samples from both the bull and the post-viremic calves revealed identical viral sequences, implicating the resident bull as the likely source of infection. Notably, the Cincinnati strain shares genetic similarity with a strain associated with a recent fatal case in a Swiss zoo (Umesh) (22) (Table 1).

**TABLE 1:** DNA sequence comparisons between EEHV1A strains^*e*^

Gene/CaseName	Kimba^*a*^	Cincinnati^*b*^	Umesh^*c*^
U38/POL (CD-1)	A	A	A
U71/gM	A	A	A
U51/vGPCR	A	E^*d*^	E
TK-gH	A2	D	D
U39/gB (CD-1)	A1	C	C1
U47/gO (CD-II)	C	C	A
U48/gH (CD-II)	A2	D	D
U82/gL (CD-III)	A1	A2	A1

^*a*^ Vaccine strain (GenBank: KC618527).

^*b*^ Challenge strain.

^*c*^ Ackermann et al. (22) (GenBank: OR543011).

^*d*^ Partial sequencing of this locus was unable to resolve whether it belongs in the subtype category of E/A.

^*e*^ Strain designations were assigned as described previously (21, 22, 24, 25).

**Fig 5:**
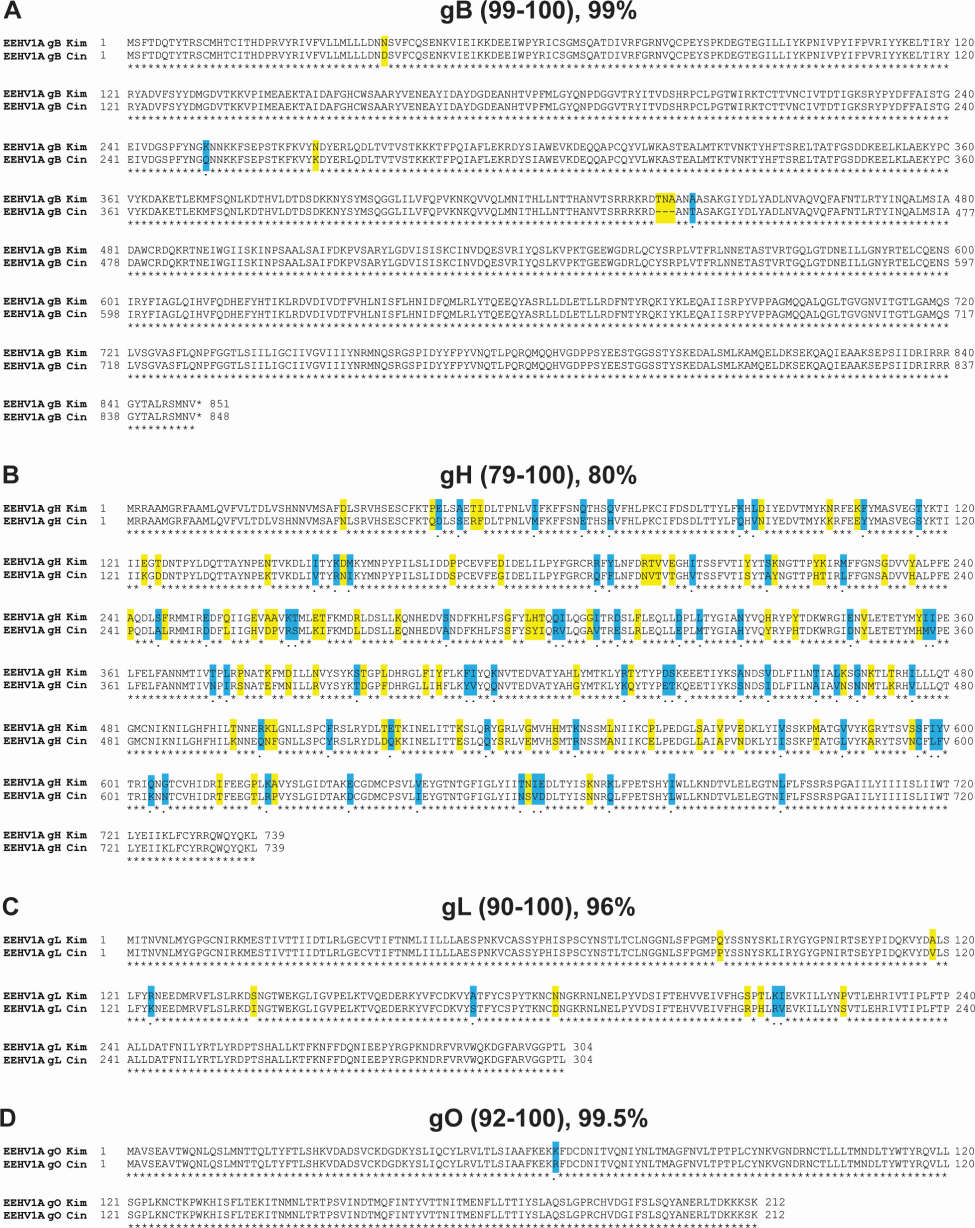
Alignments of the vaccine strain (Kimba) gB, gH, gL, and gO glycoproteins with the homologous glycoproteins from the Cincinnati EEHV1A strain. (**A**) Amino acid alignments for gB, (**B**) amino acid alignments for gH, (**C**) amino acid alignments for gL, and (**D**) amino acid alignments for gO. Amino acid residues highlighted in yellow indicate similar changes, while those highlighted in blue indicate non-similar changes. The range of divergence for each glycoprotein among EEHV1A strains is indicated above each comparison, and the level of divergence between the Cincinnati strain and Kimba is indicated.

Complete sequencing of the four glycoproteins included in the vaccine demonstrated high sequence identity for gB, gL, and gO (Fig. 5). However, gH exhibited only 80% similarity to the vaccine strain and clustered with the most genetically divergent gH sequences relative to this strain (21).

### Outcome and follow-up

**Fig 6:**
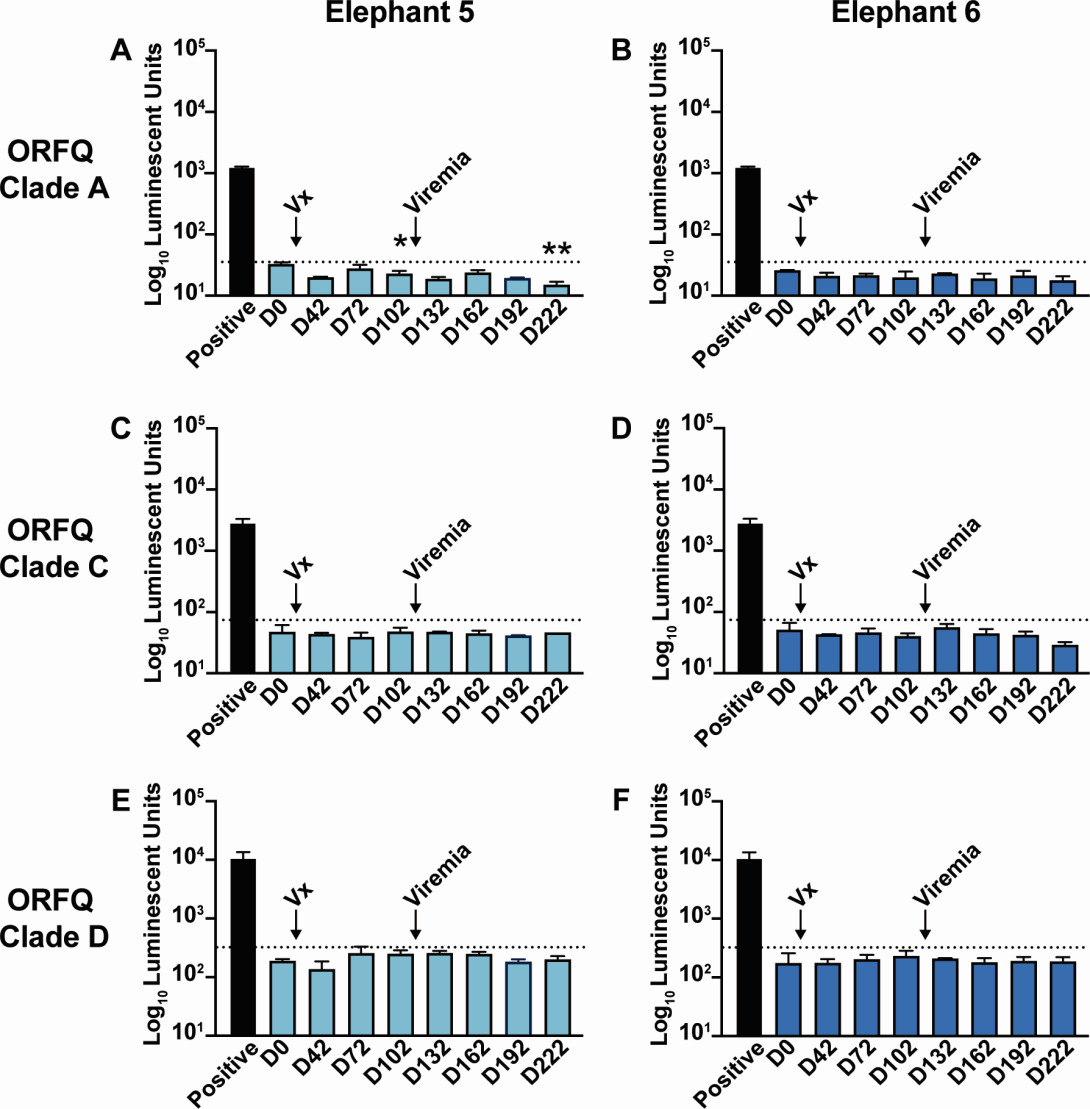
Antibody response to EEHV1A ORFQ loci before and after infection. Detection of anti-ORFQ antibodies from clade A (**A and B**), clade C (**C and D**), and clade D (**E and F**) by LIPS assay. A positive control from an elephant with prior infection with the corresponding strain was included with each experiment. Arrows indicate approximate timing of vaccinations and onset of EEHV1A viremia. Values shown are two technical replicates and are representative of at least two independent experiments. Significance relative to day 0 (D0) was identified through a repeated measures one-way ANOVA. *P < 0.05, **P < 0.01.

Both elephants experienced primary EEHV1A infection within 5 months of vaccination but remained asymptomatic and did not require treatment. Post-viremia, antibody levels against gH, gO, and the trimeric complex continued to rise, exceeding levels achieved through vaccination alone for some of the vaccine antigens (Fig. 2). In addition to viremia and shedding data, seroconversion against a viral protein expressed by the virus but not included in the vaccine was investigated. One biomarker useful for such analyses is ORFQ. At present, antibodies against ORFQ remained below the detection threshold (Fig. 6). However, previous studies suggest that seroconversion to ORFQ may take up to 1 year following primary EEHV1A infection (26) (unpublished observations). At the time of writing, viremia in both elephants has resolved. However, intermittent EEHV1A shedding continues to be detected in trunk wash samples.

## DISCUSSION

A critical question regarding the multiantigen EEHV1A mRNA vaccine is whether it can elicit protective immunity in elephants whose EEHV1A serostatus predicts a poor outcome following primary infection. Here, we provide evidence that two seronegative juvenile elephants vaccinated with the multiantigen EEHV1A mRNA vaccine 4–5 months prior to exposure survived primary infection without clinical illness or the need for medical intervention. This protection was associated with sustained antibody responses against antigens encoded by the vaccine (Fig. 2) and a low-level EEHV1A viremia (<6,000 VGE/mL) that persisted for 6–8 weeks before clearing spontaneously (Fig. 4A and B). Hematological values did not exceed 20% of the calculated reference range for more than three consecutive readings (Fig. 4C and D). Although three of the infecting viral strain’s encoded glycoproteins are closely related to the vaccine reference strain, its glycoprotein H was among the most divergent variants compared to the vaccine strain (Fig. 5). These findings indicate that the multiantigen EEHV1A mRNA vaccine can confer protection against disease caused by primary EEHV1A infection, even when the infecting strain exhibits substantial glycoprotein divergence from the vaccine strain.

Currently, no established correlate of protection (e.g., antibody levels) exists for EEHV1A infection. However, emerging evidence provides insight into the potential role of antibodies. Newborn calves possess high levels of maternal antibodies that persist for up to 2–3 years (12, 13, 15, 16), whereas the age range for EEHV-HD onset spans 2–8 years (1). This pattern suggests that maternal antibodies, particularly at high titers, may confer partial or full protection during the first 1–2 years of life. In the accompanying study, antibody responses to trimeric glycoproteins gH/L/O in vaccinated elephants approached levels observed in some EEHV1A-experienced individuals (19). One hypothesis is that these antibody levels were sufficient to blunt initial EEHV1A replication, allowing time for the development of an effective cellular immune response, known to be essential for herpesvirus control and clearance in other species (28–36). A second, non-mutually exclusive possibility is that the mRNA vaccine also induced cellular immunity and/or memory responses that, together with pre-existing antibodies, provided robust protection against severe disease. We have previously shown that the mRNA vaccine is sufficient to induce a strong cellular response in mouse studies (20), particularly against gB, consistent with our earlier finding that gB is the most immunoprevalent antigen recognized by T cells in chronically infected adult elephants (37).

Primary EEHV1A infection in seronegative elephants, or those with waning maternal antibodies, is typically characterized by marked alterations in leukocyte differentials, including lymphopenia, monocytopenia, and thrombocytopenia. High viral loads, often exceeding 100,000 viral genome equivalents per milliliter (VGE/mL) (and frequently surpassing 1,000,000 VGE/mL), are strongly associated with severe clinical illness or mortality (1, 10, 12, 18, 26). In such cases, viremia generally rises rapidly to peak levels in a few days and, in survivors, declines gradually over several days, although intermittent fluctuations above and below the assay’s limit of detection may persist for weeks to months. The cases presented here are unusual in that primary EEHV1A infection occurred in elephants with partial immunity induced by vaccination. The basis for the biphasic viremia observed in these vaccinated animals remains uncertain; however, the magnitude of viremia was substantially lower than that seen in clinical or lethal cases (<6,000 VGE/mL). A similar biphasic profile has been described for murine cytomegalovirus (mCMV), another betaherpesvirus (38, 39). In the mCMV model, circulating mononuclear leukocytes disseminate virus from the initial inoculation site, generating an early wave of viremia that diminishes as replication intensifies within reticuloendothelial tissues such as the liver and spleen. A second, more pronounced viremic phase follows, driven by renewed infection of circulating leukocytes. Evidence from fatal EEHV cases indicates that the virus is tropic for monocytes and accumulates at high levels within reticuloendothelial organs (2, 40). These parallels raise the possibility that EEHV infection may follow a similar trajectory of initial leukocyte-mediated dissemination, followed by secondary amplification. In vaccinated elephants, however, pre-existing immunity may dampen the magnitude of this secondary wave, producing the attenuated biphasic pattern observed in this study. Fluctuations in monocyte and heterophil counts appeared to loosely parallel changes in viral load, suggesting that leukocyte dynamics may contribute to the observed pattern of viremia. One potential explanation is that direct viral infection and destruction of mononuclear cells trigger transient increases in circulating virus and associated inflammatory responses, accompanied by rises in heterophil counts. It is also possible that multiple mechanisms operate simultaneously.

gB proteins encoded by all Asian elephant EEHV types are relatively well conserved and share numerous cross-reactive epitopes with other EEHV types, as demonstrated in various serology assays (7, 12, 15, 16). Thus, prior infection with other EEHV types might be predicted to provide at least some cross-protective immunity, especially against gB. However, several studies indicate that prior infection with one or more EEHV types other than EEHV1A does not appear to be sufficient to protect from EEHV1A-associated disease (10, 12, 13, 15, 16). Thus, EEHV1A-specific epitopes within gB may still be required for optimal protection mediated by antibodies or cellular responses. At present, practical challenges in obtaining high-quality peripheral blood lymphocytes (PBMCs) from young calves for assessing cellular responses limit our ability to fully evaluate cellular responses post-vaccine or post-viremia.

EEHV1A strains display significant hypervariability across several loci, particularly within the gH gene (21, 23, 25), raising concerns about whether the vaccine strain is sufficiently representative to induce protective immunity against more divergent 1A strains. In this study, gH clustered close to some of the most divergent gH genes from the vaccine strain, suggesting that the vaccine can induce broadly protective immunity against other 1A strains. However, it remains to be determined whether diverged gL and/or gO proteins expressed in other 1A strains, which are far less variable among 1A strains, could still contribute to immune escape from vaccine-induced immunity. Anecdotally, we are unaware of any evidence that elephants previously infected with EEHV1A develop illness upon reinfection with more divergent 1A strains, suggesting that prior infection with EEHV1A likely confers broad protection against disease caused by other 1A variants.

There is no evidence from either LIPS or ELISA assays, both of which detect EEHV1A-specific antibodies against multiple antigens, that any significant residual anti-EEHV1A antibodies were present at the time of vaccination, which occurred three months after the second transfusion. It is also unlikely that any viable immune cells were transferred, as the plasma was collected 2–6 years previously, frozen, and thawed prior to administration. In contrast, vaccination induced robust responses against most vaccine antigens, and these responses were sustained for several weeks before natural infection. These findings support the conclusion that any contributing protective factors from the transfused plasma had likely dissipated by the time of vaccination and were almost certainly absent by the time of infection, which occurred more than 6 months after the last transfusion.

This work delivers the first worldwide evidence that an EEHV1A-specific vaccine can protect elephants against clinical illness or disease from primary EEHV1A infection. Vaccinated juveniles remained clinically healthy following natural exposure, even to a strain with significant glycoprotein divergence, highlighting the potential breadth of vaccine-induced immunity. These results mark a critical milestone for EEHV-HD prevention and open the door to broader immunization programs aimed at reducing mortality in Asian elephants. Future studies should define correlates of protection, evaluate durability across diverse EEHV1A variants, and explore integration of vaccination into global conservation strategies.

## Data Availability

Accession numbers for the sequences reported in Table 1 are as follows: U38/POL, PX925996; U71/gM, PX926002; U51/GPCR, PX926001; TK-gH, PX926000; U39/gB, PX925996; U47/gO, PX925998; U48/gH, PX925999; and U82/gL, PX926003. The data supporting the findings of this study are available from the corresponding author upon reasonable request. All data generated or analyzed during this study are included in this article.
